# *SETD2* loss sensitizes cells to PI3Kβ and AKT inhibition

**DOI:** 10.18632/oncotarget.26567

**Published:** 2019-01-18

**Authors:** Esteban A. Terzo, Aaron R. Lim, Anna Chytil, Yun Chen Chiang, Leah Farmer, Kathryn H. Gessner, Cheryl Lyn Walker, Valerie M. Jansen, W. Kimryn Rathmell

**Affiliations:** ^1^ Vanderbilt-Ingram Cancer Center, Division of Hematology and Oncology, Department of Medicine, Vanderbilt University Medical Center, Nashville 37232, TN, USA; ^2^ Medical Scientist Training Program, Vanderbilt University School of Medicine, Nashville 37232, TN, USA; ^3^ Lineberger Comprehensive Cancer Center, University of North Carolina, Chapel Hill 27599, NC, USA; ^4^ Center for Precision Environmental Health, Baylor College of Medicine, Houston 77030, TX, USA; ^5^ Current Address: Constellation Pharmaceuticals, Cambridge, MA, USA; ^6^ Current Address: Novella/IQVIA, Morrisville, NC, USA; ^7^ Current Address: Eli Lilly and Company, Indianapolis, IN, USA

**Keywords:** SETD2, PI3Kβ, synthetic lethality, renal cell carcinoma

## Abstract

Upregulation of the PI3K pathway has been implicated in the initiation and progression of several types of cancer, including renal cell carcinoma (RCC). Although several targeted therapies have been developed for RCC, durable and complete responses are exceptional. Thus, advanced RCC remains a lethal disease, underscoring the need of robust biomarker-based strategies to treat RCC. We report a synthetic lethal interaction between inhibition of phosphatidylinositol 3-kinase beta (PI3Kβ) and loss of *SETD2* methyltransferase. Clear cell RCC (ccRCC)-derived *SETD2* knockout 786-0 and *SETD2* mutant A498 cells treated with TGX221 (PI3Kβ-specific) and AZD8186 (PI3Kβ- and δ-specific) inhibitors displayed decreased cell viability, cell growth, and migration compared to *SETD2* proficient 786-0 cells. Inhibition of the p110 δ and α isoforms alone had modest (δ) and no (α) effect on ccRCC cell viability, growth, and migration. *In vivo*, treatment of *SETD2* mutant A498 cells, but not *SETD2* proficient 786-0 cells, with AZD8186 significantly decreased tumor growth. Interestingly, inhibition of the downstream effector AKT (MK2206) recapitulated the effects observed in AZD8186-treated *SETD2* deficient cells. Our data show that specific inhibition of PI3Kβ causes synthetic lethality with *SETD2* loss and suggest targeting of the AKT downstream effector pathway offers a rationale for further translational and clinical investigation of PI3Kβ-specific inhibitors in ccRCC.

## INTRODUCTION

Renal cell carcinoma (RCC) is composed of a heterogeneous group of cancers and is the ninth most common cancer worldwide [1]. Clear cell renal cell carcinoma (ccRCC), the most common histological subtype, accounts for the majority of RCC-related deaths [2]. ccRCC tumors are known to be unresponsive to traditional chemotherapies and lack the genetic hallmarks of other solid tumors, such as *KRAS* and *TP53* mutations [3]. A number of targeted therapies against the vascular endothelial growth factor (VEGF) and mechanistic target of rapamycin (mTOR) pathways have been developed, in addition to recent advances in immunotherapy, but the response to these treatments is varied with the majority of patients eventually developing progressive disease [4]. This underscores the urgent need to identify biomarkers that better predict tumor behavior in response to targeted therapeutics.

In ccRCC tumors, the tumor suppressor von Hippel-Lindau (*VHL*) is the most frequently mutated gene [5, 6]. Its complete inactivation by either mutation or methylation is observed in more than 80% of ccRCCs and represents the earliest truncal oncogenic event in these tumors [7–10]. In recent years, large-scale cancer genomic projects have revealed numerous additional mutations in other tumor suppressors genes encoding chromatin remodelers, including protein polybromo 1 (*PBRM1/BAF180*), BRCA associated protein 1 (*BAP1)*, and Set domain containing 2 (*SETD2*) [11–13]. As opposed to *VHL* inactivation, a known founding event of ccRCC, mutations in genes involved in disease progression such as *PBRM1*, *BAP1*, and *SETD2* are associated with aggressive clinical features [14–16].

*SETD2* encodes a methyltransferase known to be responsible for the trimethylation of lysine 36 on histone H3 (H3K36me3) [17, 18], a mark associated with actively transcribed genes. In addition to H3K36, SETD2 methylates two novel non-histone targets: α tubulin on lysine 40 (αTubK40me3) of mitotic microtubules [19] and STAT1 on lysine 525 (STAT1K525me1) [20]. By methylating such diverse targets, SETD2 contributes to the maintenance of a wide spectrum of biological processes ranging from chromatin accessibility, mRNA splicing and processing [21], DNA double-strand break repair [22], genomic stability [19], and cellular defense against viral infection [20]. The diversity of molecular pathways requiring SETD2's methylating activity underscores the enzyme's crucial role in maintaining cellular homeostasis and warrants further investigation into molecular networks involving SETD2 that drive ccRCC oncogenesis.

The phosphoinositide 3-kinase (PI3K)-AKT axis is the most commonly altered molecular pathway in cancer [23]. Although the PI3K-AKT pathway presents a relatively low overall mutation rate in ccRCC when compared to other cancer types, the overall activation of AKT and downstream substrates is high [24–26]. A recent study utilizing the Genomics of Drug Sensitivity in Cancer (GDSC) database identified that RCC cells with mutated *VHL* or *SETD2* were sensitive to the small molecule PIK3β inhibitor TGX221 [27]. TGX221 was also shown to target cancer cells with *CDKN2A* and *PTEN* mutations, suggesting nonspecific inhibition at the molar concentration (5 μM) used in the study.

In this study, we sought to expand on this reported sensitivity by examining the effects of genetic and pharmacologic inhibition of the PI3K-AKT axis and its downstream effectors in more well-defined and *in vivo* model systems. We show that *SETD2* deficient 786-0 and A498 cells are significantly more sensitive to PI3Kβ-specific (TGX221 and GSK2636) and PI3Kβ/δ-specific (AZD8186) inhibitors than *SETD2* proficient (+/+) isogenic paired 786-0 cells, as evidenced by impaired viability, cell migration, spheroid formation, as well as genotype-selective reduced growth *in vivo*. These findings are replicated with siRNA approaches to confirm the target involvement. At the molecular level, we show that pAKT-S473, pPRAS40, and pS6-S235/236 phosphorylation levels are selectively reduced in *SETD2* deficient cell lines treated with the PI3Kβ-specific inhibitors TGX221 and AZD8186. Lastly, *SETD2* deficient cell lines treated with MK2206 (AKT-specific inhibitor) recapitulated the effects observed in AZD8186-treated *SETD2* deficient cells, implicating canonical PI3K signaling via AKT as a key mechanism of viability. Combined, our data demonstrate a molecular crosstalk between SETD2 methyltransferase and PI3Kβ kinase critical for *in vitro* cell proliferation and migration and for growth *in vivo*. Further, our results demonstrate that p-AKT-S473 is an integral component of the SETD2-PI3Kβ axis and shed light on the molecular mechanism underlying this novel pathway.

## RESULTS

### PI3Kβ-specific inhibitors cause synthetic lethality with *SETD2* loss in ccRCC-derived cells

We have observed that the deletion of *SETD2*, following clonal selection, consistently produces cells with a slightly more rapid cell cycle transit time [28], and a measurable advantage in proliferation. Notably, *SETD2* knockout (KO) ccRCC-derived 786-0 cells, previously generated and described in more detail [19], showed a significantly higher proliferation rate than their *SETD2* proficient (+/+) counterparts (Supplementary Figure 1). To explore the molecular mechanism underlying the proliferative advantage of these cells and determine whether critical vulnerabilities exist between targetable PI3K-AKT pathway members and *SETD2* loss, we treated *SETD2* proficient and *SETD2* deficient ccRCC-derived cell lines with a panel of inhibitors targeting PI3Kα (BYL719); PI3Kβ (TGX221, GSK2636, AZD8186); PI3Kδ (Idelalisib); and all PI3K isoforms with a Pan-PI3K inhibitor (BKM120). In addition to 786-0 *SETD2* proficient (+/+) and *SETD2* knockout (KO) cells, we used *SETD2* deficient A498 cells, which have lost one *SETD2* allele due to loss of the short arm of chromosome 3 (3p) and carry a two-base pair c.6098_6099 deletion (delTG) that causes a frameshift in the carboxyl terminus that inactivates the second allele. These cells express a SETD2 with reduced histone H3 on lysine 36 trimethylating activity and hereinafter referred to as (–/–), for simplicity.

**Figure 1:**
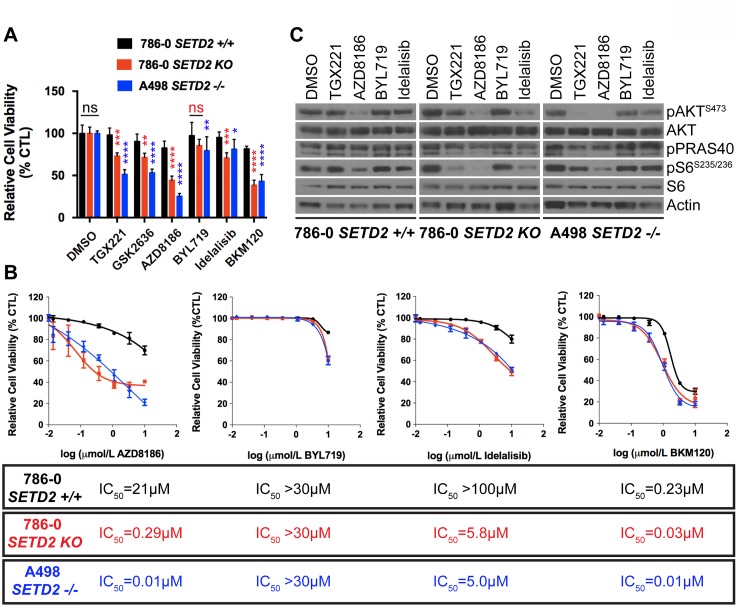
Increased sensitivity of *SETD2* deficient ccRCC-derived cells to PI3Kβ-specific inhibitors (**A**) Bar graph showing relative cell viability as a percentage of CTL (DMSO-treated) of *SETD2* proficient (+/+) 786-0 and *SETD2* deficient (KO) 786-0 and (–/–) A498 cells in response to treatment with small-molecule inhibitors. ^*^P < 0.05; ^**^P < 0.005; ^***^P < 0.001; ^****^P < 0.0001; ns, no statistical significance. Standard deviations were calculated and represented for all conditions. (**B**) Dose-response curves showing sensitivity to AZD8186, BYL719, Idelalisib, and BKM120 at different concentrations. IC_50_ was calculated for each treated cell line with a non-linear fit of transformed values using GraphPad software. (**C**) Western blot analysis of indicated proteins showing variations in phosphorylation levels in response to chemical inhibition. Whole-cell protein lysates from cells treated with 1 μM inhibitor for 24 hours were resolved by SDS-PAGE. Actin is a loading control.

We seeded all three cell lines in duplicate into 24-well plates and assessed viability by counting cell number from one well and by staining living cells with 0.3% crystal violet solution in the other for each treatment at 10 days, and obtained data from three independent biological experiments. Characterization of the three cell lines by immunoblotting showed that, as expected, *SETD2* proficient 786-0 cells express a functional SETD2 protein, whereas *SETD2* KO 786-0 cells do not. Moreover, *SETD2* (–/–) A498 cells express reduced levels of SETD2 protein accompanied by a noticeably reduced capacity to trimethylate histone H3 on lysine 36 (H3K36me3) (Supplementary Figure 2A). The two *SETD2* deficient cell lines showed a significant increase in sensitivity to all three PI3Kβ (AZD8186>TGX221>GSK2636) inhibitors, as evidenced by their decreased relative cell viability. When treated with the PI3Kα-specific inhibitor BYL719, *SETD2* deficient 786-0 and A498 lines did not show the pronounced relative cell viability changes consistently observed with PI3Kβ-specific inhibitors, although viability was somewhat decreased (Figure 1A and Supplementary Figure 2B). As AZD8186 is known to inhibit both PI3K β and δ isoforms in a cell-free assay (IC_50_ = 4 nM and 12 nM, respectively), we also treated cells with the PI3Kδ-specific inhibitor Idelalisib to see if this PI3K isoform also contributed to synthetic lethality with *SETD2* loss. Both *SETD2* deficient cell lines were sensitive to Idelalisib; however, cell viability was not as dramatically decreased as with PI3Kβ-specific inhibitors (Figure 1A and Supplementary Figure 2B). When *SETD2* deficient cells (KO 786-0 and (–/–) A498) were treated with the Pan-PI3K inhibitor BKM120, they displayed a significant increase in sensitivity that resembled the effects observed with PI3Kβ-specific inhibitors (Figure 1A and Supplementary Figure 2B).

To further examine whether the observed sensitivity of *SETD2* deficient cells was specifically determined by PI3Kβ isoform inhibition, we conducted a dose-response assay using AZD8186, as it displayed the most pronounced response among the three PI3Kβ-specific inhibitors (Figure 1A), along with BYL719 (PI3Kα-specific), Idelalisib (PI3Kδ-specific), and BKM120 (Pan-PI3K) inhibitors and calculated their respective half maximal inhibitory concentrations (IC_50_s). Dose-response curves showed that *SETD2* deficient 786-0 and A498 cells were highly sensitive to AZD8186 when compared to *SETD2* proficient 786-0 cells (IC_50_ = 0.29 μM, 0.01 μM, and 21 μM respectively) (Figure 1B). In addition, all three cell lines were only sensitive to BYL719 at toxic concentrations [29]. Idelalisib dose-response curves showed that *SETD2* deficient 786-0 and A498 cells were more sensitive to the PI3Kδ-specific inhibitor than *SETD2* proficient 786-0 cells. However, the difference was not as pronounced as that observed with AZD8186 treatment. The Pan-PI3K dose-response curves also showed that, at low concentrations, *SETD2* deficient cells were more sensitive to the compound than *SETD2* proficient cells. However, at higher drug concentrations, sensitivity was SETD2-independent, as all three cell lines showed increased sensitivity. Combined, these data show that PI3Kβ-specific inhibitors, and most prominently AZD8186, cause a synthetic lethal-type interaction with *SETD2* loss in ccRCC-derived cell lines.

### PI3Kβ-specific inhibitors TGX221 and AZD8186 decrease proliferation in *SETD2* deficient ccRCC-derived cells

To explore if the synthetic lethal interaction between *SETD2* loss and targeted PI3Kβ inhibition specifically affects cell proliferation, we treated *SETD2 (+/+)* 786-0, *SETD2* (*KO*) 786-0, and *SETD2 (–/–)* A498 cell lines with TGX221, AZD8186, and BYL719 and measured cell number over 7 days. *SETD2* deficient cells showed a significantly decreased proliferation rate when treated with TGX221 and consistently more so with AZD8186, while BYL719 showed a mild or no decrease in all tested cell lines (Supplementary Figure 2C). Notably, 786-0 *SETD2* KO cells rescued with functional truncated SETD2 (tSETD2) abrogated sensitivity to PI3Kβ inhibition, whereas a catalytically inactivating mutation in the SET domain (R1625C) still retained increased sensitivity to PI3Kβ inhibition (Supplementary Figure 2D).

To interrogate what downstream effectors of the PI3Kβ-AKT axis might mediate the synthetic lethal interaction, we conducted a drug-target engagement experiment treating *SETD2* proficient (786-0) and *SETD2* deficient (786-0 and A498) cells with 1 μM inhibitor for 24 hours. Immunoblotting of whole-cell extracts from treated cells showed that phosphorylation of pAKT-S473 and pS6-S235/236 were decreased in *SETD2* deficient cells relative to SETD2-proficient cells by TGX221, whereas AZD8186 treatment resulted in inhibition of AKT and S6 phosphorylation in both SETD2-proficient and SETD2-deficient cells. The same was observed for pPRAS40, which is a phosphorylation target of AKT (Figure 1C). These results show that *SETD2* deficient ccRCC-derived cells are significantly less proliferative when treated with PI3Kβ inhibitors and strongly suggest inhibition of downstream effectors in the PI3K pathway (pAKT-S473 and pS6) may play a role in this synthetic lethal interaction.

### Genetic inhibition of PI3Kβ with siRNA reduces viability of *SETD2* deficient ccRCC-derived cells

**Figure 2:**
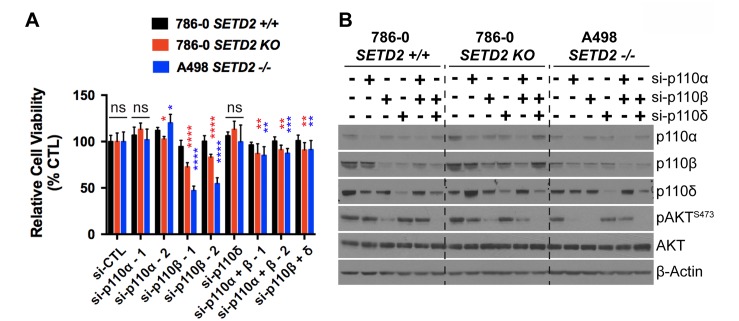
Genetic inhibition of PI3K p110β with siRNA reduces viability of *SETD2* deficient ccRCC-derived cells (**A**) Bar graph showing relative cell viability as a percentage of control (si-CTL) of *SETD2* (+/+) 786-0 and *SETD2* (KO) 786-0 and (–/–) A498 cells in response to treatment with siRNA specific to p110α, p110β, and p110δ (singlets and doublets). ^*^P < 0.05; ^**^P < 0.005; ^***^P < 0.001; ^****^P < 0.0001; ns, no statistical significance observed. Standard deviations were calculated and represented for all conditions. (**B**) Western blot analysis of indicated proteins showing decrease in expression of p110α, p110β, and p110δ in individual or combinatorial treatments (specificity) and variations in phosphorylation levels of known downstream target pAKT-S473 (efficacy). Actin is a loading control.

To confirm that pharmacological inhibition (small-molecule inhibitors) specifically causes synthetic lethality in *SETD2* deficient cells via effects on PI3Kβ (as opposed to off-target effects elsewhere), we conducted a genetic knockdown experiment using siRNA specifically targeting the α, β, and δ isoforms of PI3K. We interrogated the effect of siRNA for single targets or in combinations (α/β and β/δ) on cell viability. We predicted that siRNA targeting PI3Kβ would closely replicate the effect on relative cell viability observed in *SETD2* deficient ccRCC-derived cells when treated with PI3Kβ-specific inhibitors. Knockdown using two different siRNAs specifically targeting PI3Kβ (si-p110β-1 and -2) consistently showed a significant decrease in the relative viability of both *SETD2* KO 786-0 and *SETD2* –/– A498 cells when compared to that of *SETD2* proficient 786-0 cells, a phenomenon not observed with si-p110α- or si-p110δ-specific knockdowns (Figure 2A); immunoblotting confirmed the specificity of the single and combinatorial knockdowns (Figure 2B).

When siRNAs targeting PI3Kβ were used in combination with those against α and δ PI3K isoforms, the combinatorial siRNA treatments showed a significant decrease in relative cell viability, but not as pronounced as the effect observed when cells were treated with either of these siRNAs against PI3Kβ (si-p110β-1 or -2) alone (Figure 2A). Phosphorylation levels of a PI3K downstream effector, pAKT-S473, were assessed by immunoblot analysis to corroborate the efficiency of PI3K isoform knockdowns. We found that reduced levels of pAKT-S473 were observed in whole cell extracts from the three RCC-derived cell lines when treated with siRNA against PI3Kβ alone and in combination with si-p110δ (Figure 2B). These results strongly suggest that the decrease in pAKT-S473 phosphorylation levels observed when PI3Kβ is chemically inhibited (TGX221 and more consistently AZD8186) (Figure 1C) was due to the specific inhibition of PI3Kβ's enzymatic activity, revealing pAKT-S473 as a critical reporter, and perhaps mediator, of the SETD2-dependency on the PI3Kβ signaling network.

### PI3Kβ-specific inhibitors TGX221 and AZD8186 abrogate spheroid formation and cell migration in *SETD2* deficient ccRCC-derived cells

**Figure 3:**
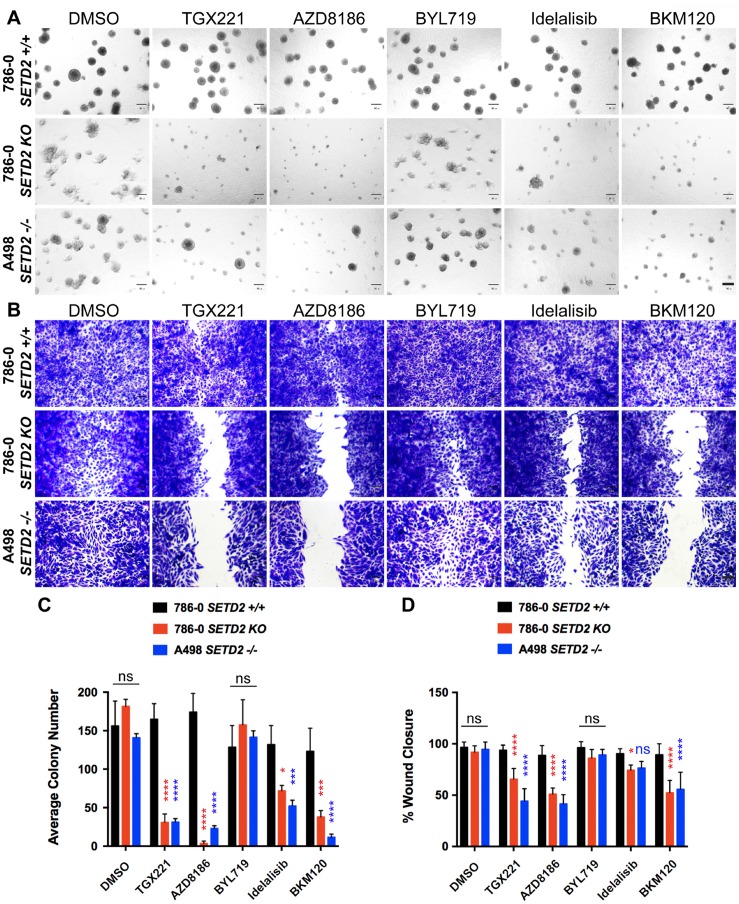
PI3Kβ-specific inhibitors TGX221 and AZD8186 abrogate spheroid formation and cell migration in *SETD2* deficient ccRCC-derived cells (**A**) Phase-contrast pictures showing spheroid formation of *SETD2* (+/+) 786-0 and *SETD2* (KO) 786-0 and (–/–) A498 cells in response to treatment with vehicle (DMSO) and 500 nM inhibitor for 14 days grown in Matrigel. Scale bar: 30 μm. (**B**) Bright-field microscopy images showing living cells stained with 0.3% crystal violet solution. Treated cells (1 μM for 3 days) incapable of migrating show remnants of scratch (wound) of different widths. Scale bar: 30 μm. (**C**) Bar graph showing average colony number for the three cell lines treated with different inhibitors. ^*^P < 0.05; ^***^P < 0.001; ^****^P < 0.0001; ns, no statistical significance observed. Standard deviations were calculated and represented for all conditions. (**D**) Bar graph showing percentage (%) of wound closure compared to cells treated with vehicle (DMSO). ^*^P < 0.05; ^****^P < 0.0001; ns, no statistical significance observed. Standard deviations were calculated and represented for all conditions.

To determine if the tumor growth capacity of *SETD2* deficient ccRCC-derived cell lines was similarly dependent on PI3Kβ, we utilized 3-D spheroid cultures in Matrigel to allow cells to self-assemble into organotypic structures (spheroids). These spheroids mimic *in vitro* tumor morphology adopted *in vivo* and provide a tractable model for evaluating tumor growth in response to pharmacological agents. Untreated *SETD2* proficient 786-0 cells displayed a round spheroid morphology that remained almost unaffected despite the diverse treatments with PI3K isoform-specific and Pan PI3K inhibitors (Figure 3A). Conversely, both *SETD2* deficient cell lines showed a less structured spheroid morphology and a significant decrease in their number when treated with PI3Kβ-specific, but not PI3Kα-specific, inhibitors when compared to *SETD2* proficient cells (Figure 3A and 3C). Interestingly, both Idelalisib (PI3Kδ-specific) and BKM120 (Pan-PI3K) inhibitors showed significant cell growth decrease in *SETD2* deficient cell lines, but more prominently with the latter inhibitor, recapitulating the effects observed with PI3Kβ-specific inhibitors (Figure 3C).

To interrogate the ability of *SETD2* deficient cells to migrate in the presence of pharmacological agents, we performed a 2-D wound-healing assay. We then tracked migration through time (2 days) and stained with crystal violet once cells treated with vehicle reached confluency and calculated migration as a percentage (%) of wound closure. We observed that *SETD2* deficient cells were significantly less migratory than their *SETD2* proficient counterparts when treated with PI3Kβ-specific inhibitors, a phenomenon closely resembled by the treatment with the Pan-PI3K inhibitor, BKM120 (Figure 3B and 3D). Combined, these data demonstrate that the capacity of *SETD2* deficient ccRCC-derived cell lines to form spheroids (3-D) and to migrate (2-D) is significantly hindered by PI3Kβ-specific inhibitors, which we observed is closely replicated when these cells are treated with the Pan-PI3K inhibitor BKM120. These data also strongly suggest that the PI3Kβ pathway may participate in the regulation of both migration and tumor growth in SETD2-deficient states.

### PI3Kβ-specific inhibitor AZD8186 abrogates *SETD2* deficient tumor formation *in vivo*

**Figure 4:**
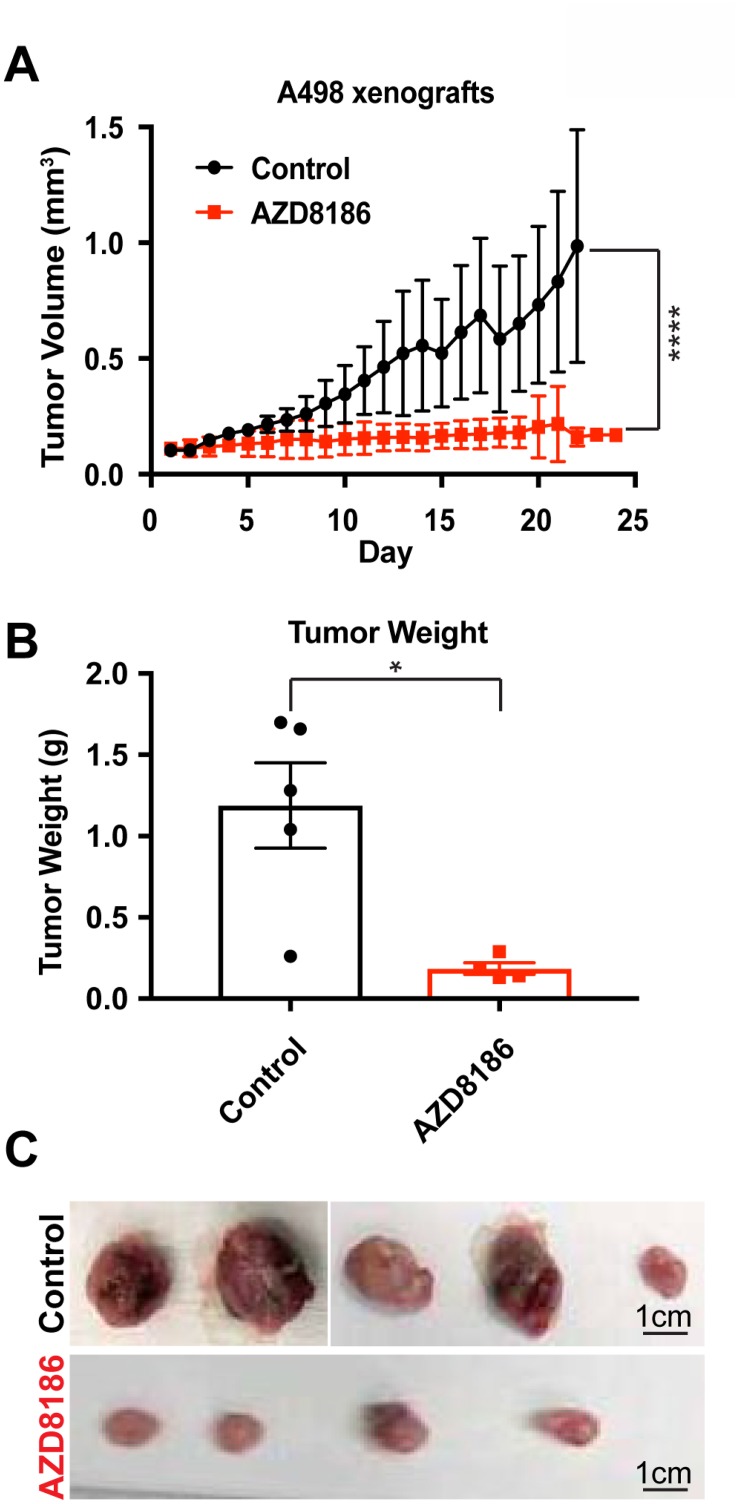
PI3Kβ-specific inhibitor AZD8186 hinders *SETD2*-deficient tumors *in vivo* (**A**) Graph plotting tumor volume (mm^3^) as a function of time (day) showing changes in volume of untreated (control) and AZD8186-treated tumors. ^****^P < 0.0001. (**B**) Bar graph showing average tumor weight (g) for control and AZD8186-treated tumors. ^*^P < 0.05. (**C**) Pictures of untreated (control) and AZD8186-treated tumors. Scale bar: 1 cm.

To translate our *in vitro* findings that *SETD2* deficient ccRCC-derived cells exhibit increased sensitivity to PI3Kβ-specific inhibition to the *in vivo* setting, we conducted xenograft studies. NOD *scid* gamma mice were injected subcutaneously with *SETD2* mutant ccRCC-derived A498 cells and approximately a month later, mice bearing tumors measuring ≥150 mm^3^ were randomized to treatment with vehicle (control) or AZD8186. Animals treated with the PI3Kβ-specific inhibitor AZD8186 showed a significant decrease in tumor growth and final tumor weight compared to control mice (Figure 4A–4C). However, 786-0 *SETD2* proficient xenograft tumors did not have a significant response to PI3Kβ inhibition (Supplementary Figure 3). Thus, AZD8186 can effectively reduce growth of *SETD2* mutant tumors but not *SETD2* wild-type tumors, underscoring the importance of the molecular connection between the PI3Kβ signaling network and *SETD2* loss as a promising therapeutic target for ccRCC.

### AKT-specific inhibitor MK2206 decreases cell viability, spheroid formation, and migration of *SETD2* deficient ccRCC-derived cells

**Figure 5:**
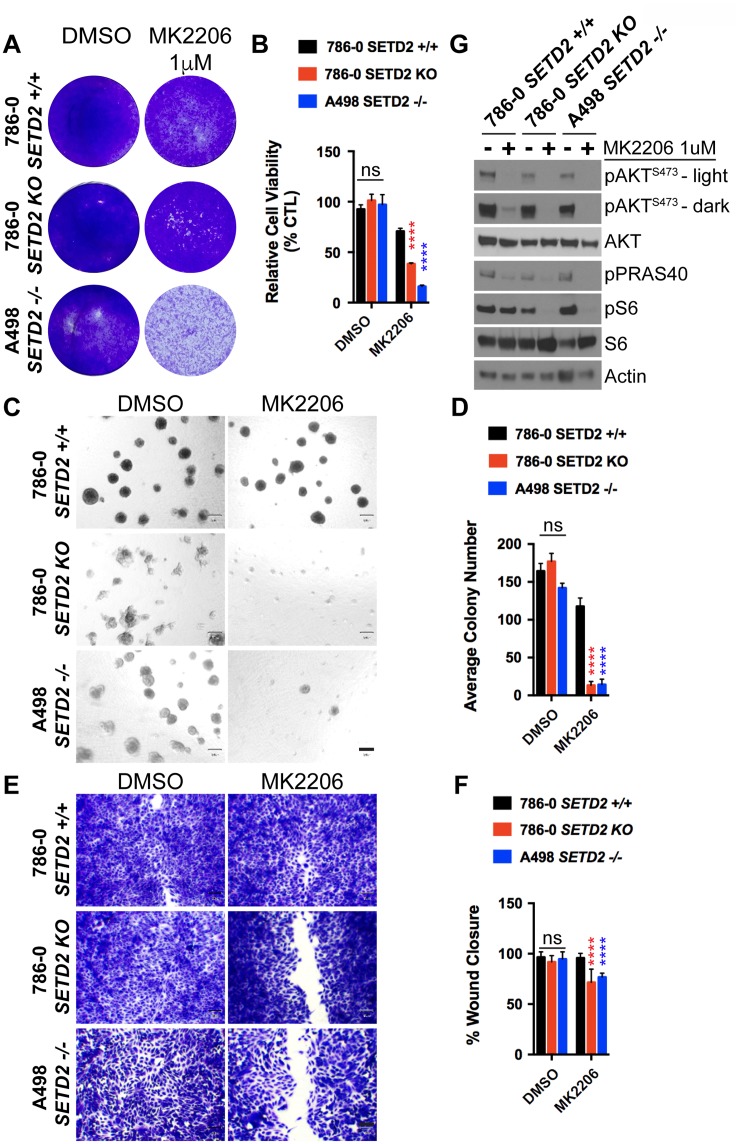
AKT-specific inhibitor MK2206 decreases cell viability, spheroid formation, and migration of *SETD2* deficient ccRCC-derived cells (**A**) Bright-field microscopy images showing living cells (attached to bottom of well) stained with 0.3% crystal violet solution of *SETD2* (+/+) 786-0 and *SETD2* (KO) 786-0 and (–/–) A498 cells were treated with vehicle (DMSO) or 1 μM AKT-specific inhibitor MK2206 for 10 days. (**B**) Bar graph showing relative cell viability as a percentage of CTL of *SETD2* (+/+) 786-0 and *SETD2* (KO) 786-0 and (–/–) A498 cells in response to treatment with MK2206. ^****^P < 0.0001; ns, no statistical significance observed. Standard deviations were calculated and represented for all conditions. (**C**) Phase-contrast pictures showing spheroid formation of *SETD2* (+/+) 786-0 and *SETD2* (KO) 786-0 and (–/–) A498 cells in response to treatment with vehicle (DMSO) and 500 nM inhibitor for 14 days grown in Matrigel. (**D**) Bar graph showing average colony number of *SETD2* (+/+) 786-0 and *SETD2* (KO) 786-0 and (–/–) A498 cells treated with MK2206 inhibitor. ^****^P < 0.0001; ns, no statistical significance observed. Standard deviations were calculated and represented for all conditions. (**E**) Bright-field microscopy images showing living cells stained with 0.3% crystal violet solution. (**F**) Bar graph showing percentage (%) of wound closure compared to cells treated with vehicle (DMSO). (**G**) Western blot analysis of indicated proteins showing variations in phosphorylation levels in response to chemical inhibition with MK2206 (AKT-specific). Whole-cell protein lysates from cells treated with 1 μM inhibitor for 24 hours were resolved by SDS-PAGE. Actin is a loading control.

Our previous data demonstrating that PI3Kβ-specific inhibitors decrease phosphorylation levels of key downstream effectors in the PI3K-AKT pathway (pAKT-S473 and pS6-S235/236) in *SETD2* deficient ccRCC-derived cells prompted us to further explore the molecular mechanism connecting SETD2 to PI3Kβ. To do this, we challenged *SETD2* proficient and *SETD2* deficient ccRCC-derived cell lines with the AKT-specific inhibitor MK2206 and observed their sensitivity to the compound, spheroid formation, and migration. Similar to the treatment with PI3Kβ-specific inhibitors, we observed that *SETD2* deficient cells were more sensitive to MK2206, as evidenced by a significant decrease in their cell viability (Figure 5A and 5B). Sensitivity of *SETD2* deficient cells was also observed in 3-D growth and 2-D wound healing assays, where they displayed a significantly reduced ability to form spheroids and to migrate (Figure 5C–5F). Immunoblotting of whole-cell lysates from a drug-target engagement assay using 1 μM of MK2206 showed that MK2206 effectively reduces phosphorylation levels of pAKT-S473. Interestingly, however, dramatically reduced pS6 phosphorylation levels were only observed in *SETD2* deficient cell lines (Figure 5G). Together, these data demonstrate that AKT is a key effector of a molecular axis connecting SETD2 to PI3Kβ that is required for the regulation of growth and migration.

## DISCUSSION

ccRCC tumors are highly lethal and are characterized by resistance to chemotherapy, resulting in targeted therapies and immunotherapy emerging as the major mechanisms for treatment. Approximately 30% of ccRCC patients presenting with localized disease develop metastases after nephrectomy [2, 30]. During the past decade, several therapeutic treatments for RCC have been developed, but treatment response is varied, and development of resistance is common, which underscores the urgent need to develop a broader panel of effective therapies for ccRCC. Here, we report a synthetic lethal interaction between targeted PI3Kβ-AKT axis and loss of *SETD2* both *in vitro* (ccRCC-derived cells) and *in vivo* (ccRCC cell line-derived xenografts).

In ccRCC tumors, *SETD2* is ubiquitously haploinsufficient (>95%) as a consequence of the loss of the short of arm of chromosome 3 (3p), a phenomenon widely accepted as an early event in ccRCC transformation [5]. *SETD2* bi-allelic loss occurs in at least 20% of primary human RCC tumors, which is associated with more advanced disease and the lethal metastatic phenotype [31]. Further, bi-allelic loss of *SETD2*, or mutations rendering the protein catalytically inactive, result in loss of H3K36me3 in ccRCC-derived cells and tumors [21, 32, 33]. Interestingly, a study of ccRCC intratumoral heterogeneity identified distinct *SETD2* mutations across subsections of an individual tumor, suggesting a selection bias for *SETD2* mutation in the course of ccRCC development [34]. This mutation is associated with the development of metastatic disease, and the mechanisms by which this mutational event supports cellular growth have been elusive to date. Cellular signaling and the remodeling of signaling pathways likely underscores the growth advantage that emerges downstream of the nuclear and cytoplasmic process (chromatin remodeling, genomic instability, impaired DNA repair) that have been directly attributed to *SETD2* loss.

One of the more significantly activated pathways in ccRCC is the PI3K-AKT-mTOR axis [8]. Phosphatidylinositol 3-kinases (PI3Ks) are a family of lipid kinases that translate numerous environmental signals from growth factors, cytokines, and other cues into signaling pathways controlling diverse biological processes, such as cell proliferation, growth, and motility among others [35]. Multiple PI3K families are known in eukaryotes. Class IA PI3Ks are heterodimers containing a p110 catalytic subunit and a p85 regulatory subunit. The genes *PIK3CA*, *PIK3CB*, and *PIK3CD* encode three highly homologous catalytic isoforms: PI3K (p110)α, PI3K (p110)β, and PI3K (p110)δ, respectively. Of note, mainly class IA PI3Ks have been implicated in cancer [36]. Further, the PI3K-AKT axis is known to be activated by gene mutations and copy number alterations (CNAs) than any other altered molecular pathway in cancer [23]. When compared to other cancers, although the PI3K-AKT pathway presents a relatively low overall mutation rate in ccRCC, the overall activation (phosphorylation levels) of AKT and downstream substrates is high [24–26].

Supporting the critical role of the PI3K-AKT pathway in ccRCC and the emerging notion as a promising druggable target, a recent study demonstrated a connection between targeted PI3Kβ inhibition with the small-molecule inhibitor TGX221 and loss of *SETD2* [27]. In this study, ccRCC-derived cells deficient for both *VHL* and *SETD2* are significantly more sensitive to TGX221 than those deficient for *VHL* alone. They also demonstrated that TGX221-treated *VHL* and *SETD2* deficient ccRCC-derived cell lines have a significantly reduced migrating and invading capacity when compared to *VHL* deficient ccRCC-derived cell lines, suggesting a novel molecular connection between PI3Kβ and SETD2. However, the mechanism underlying the crosstalk between *SETD2* loss and the targeted PI3K-AKT axis remains unknown. Our combined data demonstrating that *SETD2* deficient ccRCC-derived cells are significantly sensitive, less migratory, and less capable of forming spheroids and that tumor formation of *SETD2* mutant xenografts is abrogated, further underscore the biological relevance of this molecular connection between SETD2 and PI3Kβ. We also reveal the critical role that a functional AKT plays in supporting growth and migration specifically in *SETD2* mutant ccRCC-derived cells. Based on our findings, we propose that in ccRCC, which typically harbors inactivating *SETD2* mutations, the PI3Kβ-AKT axis is essential for growth and migration and that when targeted, is inhibitory to cells with *SETD2* loss, therefore revealing tantalizing therapeutic applications.

## MATERIALS AND METHODS

### Cell culture and generation of *SETD2*-null human 786-0 and A498 cells

786-0 and A498 cell lines were acquired from the American Type Culture Collection (Manassas, VA, USA). RCC-derived SETD2-null 786-0 cells were generated using TAL effector nucleases targeted to exon 3 of *SETD2*, as previously described [19]. Functional truncated SETD2 (tSETD2) with wild–type or SET domain mutant (R1625C) sequences were expressed in 786-0 SETD2 deficient cells as previously described [32]. All cells undergo annual STR analysis for genetic confirmation. All cell lines were cultured in Dulbecco's Modified Eagle Medium (DMEM, Gibco/Life Technologies, Carlsbad, CA, USA) supplemented with 10% FBS (Gemini Bio-Products, West Sacramento, CA, USA), non-essential amino acids, L-glutamine, penicillin, and streptomycin. All cultures were maintained at 37° C in 5% CO2.

### Dose-response assays

Cells were seeded in DMEM 10% FBS in 96-well plates. 1000 cells per well were plated in quadruplicate for each cell line (*SETD2* proficient and KO 786-0 and *SETD2* mutant A498 cells) and allowed to attach to bottom of plates overnight. The following day, cells were treated with different concentrations of inhibitors (10 μM was the highest concentration, which was subsequently diluted 6 times at a 1:3 ratio and control wells had only DMSO) for 7 days and then fix/stained with 0.3% crystal violet solution followed by image analysis of the plates using an Odyssey Infrared Imaging System (LI-COR Biosciences). IC_50_ values were determined after double log-transformation of dose response curves as previously described [37]. Alternatively, cell viability was assayed with CellTiter Glo according to the manufacturer's instructions (Promega).

### Cell proliferation assays

Cells were seeded in DMEM 10% FBS for proliferation in two-dimensional (2D) growth assays and fixed/stained with crystal violet. 3000 cells per well were plated in quadruplicate for each cell line (*SETD2* proficient and KO 786-0 and *SETD2* mutant A498 cells) and then allowed to grow for 7 days. Each row of a 24-well plate (three rows/plate) contained a cell line and each plate represented a time point [38]. Media and inhibitors were replenished every 2 days during the 7-day assay; adherent cells were fixed/stained with 0.3% crystal violet solution followed by image analysis of 24-well plates using an Odyssey Infrared Imaging System (LI-COR Biosciences). Integrated densities from three independent experiments were calculated for all treatments and then normalized to control (DMSO-treated cells) to assess growth rate.

### Cell viability assays

Cells were seeded in DMEM 10% FBS and allowed to grow in two-dimensional (2D) viability assays and counted or fixed/stained with crystal violet as described previously [29]. 3000 cells per well were plated in quadruplicate for each cell line (*SETD2* proficient and KO 786-0 and *SETD2* mutant A498 cells) and then allowed to grow for 7–10 days in the presence of 1 μM inhibitor. Media and inhibitors were replenished every 2–3 days; after 7–10 days, adherent cells were trypsinized and counted using a Coulter Counter or fixed/stained with 0.3% crystal violet solution followed by image analysis of the plates using an Odyssey Infrared Imaging System (LI-COR Biosciences). Cell number (Coulter Counter) and cell density (Crystal Violet fix/stain) for all treatments were obtained from three independent experiments and normalized to control (DMSO-treated cells) to assess cell response to inhibitors.

### siRNA transfections

Cells were transfected with human SMART pool ON-TARGETplus PIK3CB (L-003019-00-0005), PIK3CA (L-003018-00-0005), PIK3CD (L-006775-00-0005), and ON-TARGETplus Non-targeting pool (D-001810-10-05) siRNAs using DharmaFECT 1 transfection reagent (T-2-001-02) (GE Dharmacon) or with human PI3-Kinase p110α (sc-39127), PI3-Kinase p110β (sc-37269), PI3-Kinase p110δ (sc-39101), and Control siRNA-A (sc37007) siRNAs (Santa Cruz Biotechnology) using Lipofectamine RNAiMax Transfection Reagent (Invitrogen) according to the manufacturer's protocol. Transfections were performed in serum-reduced, antibiotic-free DharmaFECT Cell Culture Reagent (DCCR) (B-004500-100) (GE Dharmacon).

### 3D growth assays

3D growth assays were conducted in growth factor-reduced Matrigel (BD Biosciences) as described previously [39]. In brief, cells were resuspended in a 1:1 ratio in 5% Matrigel containing medium supplemented with the drug treatments. For each drug condition, cell mixture (400 mL of cell mixture containing 5000/cells and 500 nM of TGX221, AZD8186, BYL719, BKM120, Idelalisib, MK2206, or DMSO) was plated in triplicate wells of a 48-well plate containing solidified Matrigel. Fresh media containing 5% Matrigel and drugs (500 nM) or DMSO were replaced every 2–3 days. After 14 days, phase-contrast pictures were taken using an Olympus CK40 microscope and colonies were counted using the GelCount scanning software.

### Scratch assays

Cells were seeded in triplicate in 24-well plates such that, after 24 h of growth, cells would be ~70–80% confluent as a monolayer (25,000 cells/well for *SETD2* proficient and KO 786-0 cells; 50,000 cells/well for *SETD2* mutant A498). The following day, a “wound” was made with a new 1 ml pipette tip across the center of the well. A straight line was scratched in one direction and another straight line perpendicular to the first line to create a cross in each well. After scratching, each well was washed twice with medium to remove the detached cells and replenished with fresh medium containing drugs (1 μM). After 48 h of cell growth, cells were fixed and stained with 20% methanol/80% water/0.5% crystal violet for 30 min, washed with water, and dried. Photos for the original wound and for the stained monolayer were taken using an Olympus CK40 microscope. Gap distances from images were measured in 3 different areas in each well. The percent wound closure was calculated from the ratio of the current wound area to the original wound area. Please refer to the following link https://bio-protocol.org/e100 for more details.

### Immunoblot analysis

Adherent cells were first washed with 1× PBS, then thoroughly dried, and consequently frozen down at –20° C overnight. The day after, frozen cells were scraped and lysed with RIPA buffer (150 mM NaCl, 1.0% IGEPAL^®^, 0.5% sodium deoxycholate, 0.1% SDS, and 50 mM Tris, pH 8.0. [Sigma], and 1× protease inhibitor cocktail [Roche]). Lysates (20 μg) were resolved by SDS-PAGE and transferred to nitrocellulose or PVDF membranes; these were first incubated with primary antibodies at 4° C overnight, followed by incubation with HRP-conjugated anti-rabbit or anti-mouse secondary antibodies (1:10,000) (Santa Cruz Biotechnology) for 1 h at room temperature. Immunoreactive bands were visualized by enhanced chemiluminescence (Thermo Scientific). Antibodies and dilutions: phospho-AKT-S473 (Cell Signaling 9271, 1:500), AKT (Cell Signaling 9272, 1:1500), phospho-PRAS40 (Cell Signaling 2997, 1:500), phospho-S6-S235/236 (Cell Signaling 4858, 1:500), S6 (Cell Signaling 2217, 1:1500), β-Actin (Cell Signaling 4970, 1:5000), SETD2 (Sigma HPA042451, 1:500), Histone H3K36me3 (Active Motif 61101, 1:500), Histone H3 (Abcam ab10799, 1:500), p110α (Cell Signaling 4249, 1:500), p110β (Cell Signaling 3011, 1:500), p110δ (Cell Signaling 34050, 1:500).

### Xenograft studies

Mouse experiments were approved by the Vanderbilt Institutional Animal Care and Use Committee. Female NOD *scid* gamma (NSG) mice (Jackson Laboratories) were used. A498 or 786-0 cells (1 × 10^7^) were suspended in DMEM and Matrigel (BD Biosciences, San Jose, CA, USA) at 1:1 ratio and injected subcutaneously (s.c.) into the right flank of each mouse. Approximately 4 weeks later, mice bearing tumors measuring ≥150 mm^3^ were randomized to treatment with 1) vehicle (control) or 2) AZD8186 (30 mg/kg/day via orogastric gavage). Animal weights (data not shown) and tumor diameters (with calipers) were measured twice weekly and tumor volume was calculated with the formula: volume = (width^2^ × length)/2.

### Statistical analyses

Unless otherwise indicated, significant differences (*p* < 0.05) were determined by ANOVA using GraphPad Prism software.

## SUPPLEMENTARY MATERIALS AND FIGURES


