# Correction: *SETD2* loss sensitizes cells to PI3Kβ and AKT inhibition

**DOI:** 10.18632/oncotarget.27288

**Published:** 2019-10-29

**Authors:** Esteban A. Terzo, Aaron R. Lim, Anna Chytil, Yun Chen Chiang, Leah Farmer, Kathryn H. Gessner, Cheryl Lyn Walker, Valerie M. Jansen, W. Kimryn Rathmell

**Affiliations:** ^1^ Vanderbilt-Ingram Cancer Center, Division of Hematology and Oncology, Department of Medicine, Vanderbilt University Medical Center, Nashville 37232, TN, USA; ^2^ Medical Scientist Training Program, Vanderbilt University School of Medicine, Nashville 37232, TN, USA; ^3^ Lineberger Comprehensive Cancer Center, University of North Carolina, Chapel Hill 27599, NC, USA; ^4^ Center for Precision Environmental Health, Baylor College of Medicine, Houston 77030, TX, USA; ^5^ Current Address: Constellation Pharmaceuticals, Cambridge, MA, USA; ^6^ Current Address: Novella/IQVIA, Morrisville, NC, USA; ^7^ Current Address: Eli Lilly and Company, Indianapolis, IN, USA; ^*^ Co-first authors; ^#^ Co-corresponding authors

**This article has been corrected:** An image duplication error occurred in Figure 5E, in which a selected image was inadvertently pasted in the incorrect panel. The proper Figure 5E is shown below. The authors declare that these corrections do not change the results or conclusions of this paper.

Original article: Oncotarget. 2019; 10:647–659. 647-659. https://doi.org/10.18632/oncotarget.26567

**Figure 5:**
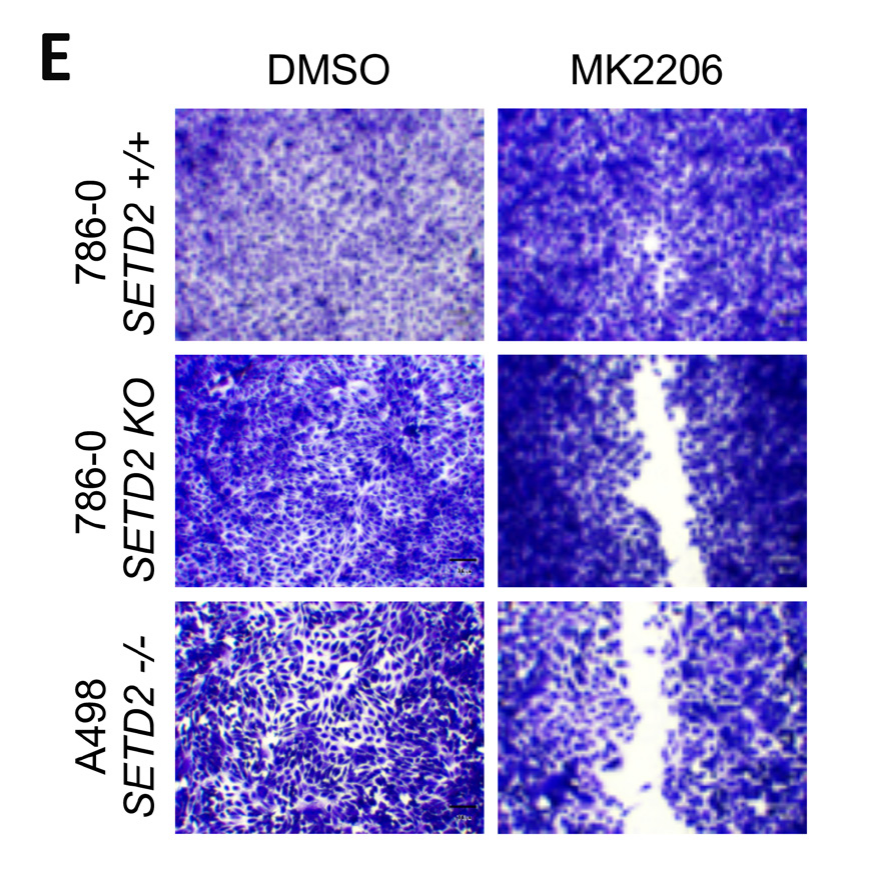
AKT-specific inhibitor MK2206 decreases cell viability, spheroid formation, and migration of SETD2 deficient ccRCC-derived cells. (**A**) Bright-field microscopy images showing living cells (attached to bottom of well) stained with 0.3% crystal violet solution of *SETD2* (+/+) 786-0 and *SETD2* (KO) 786-0 and (–/–) A498 cells were treated with vehicle (DMSO) or 1 μM AKT-specific inhibitor MK2206 for 10 days. (**B**) Bar graph showing relative cell viability as a percentage of CTL of *SETD2* (+/+) 786-0 and *SETD2* (KO) 786-0 and (–/–) A498 cells in response to treatment with MK2206. ****P < 0.0001; ns, no statistical significance observed. Standard deviations were calculated and represented for all conditions. (**C**) Phase-contrast pictures showing spheroid formation of SETD2 (+/+) 786-0 and *SETD2* (KO) 786-0 and (–/–) A498 cells in response to treatment with vehicle (DMSO) and 500 nM inhibitor for 14 days grown in Matrigel. (**D**) Bar graph showing average colony number of *SETD2* (+/+) 786-0 and *SETD2* (KO) 786-0 and (–/–) A498 cells treated with MK2206 inhibitor. ****P < 0.0001; ns, no statistical significance observed. Standard deviations were calculated and represented for all conditions. (**E**) Bright-field microscopy images showing living cells stained with 0.3% crystal violet solution. (**F**) Bar graph showing percentage (%) of wound closure compared to cells treated with vehicle (DMSO). (**G**) Western blot analysis of indicated proteins showing variations in phosphorylation levels in response to chemical inhibition with MK2206 (AKT-specific). Whole-cell protein lysates from cells treated with 1 μM inhibitor for 24 hours were resolved by SDS-PAGE. Actin is a loading control.

