# Supplementary material for: SETD2 loss sensitizes cells to PI3Kβ and AKT inhibition
Source: Oncotarget. 2019 Jan 18;10(6):647–59. doi: 10.18632/oncotarget.26567 (PMC6363018; doi:10.18632/oncotarget.26567)
Supplement: Supplementary file 1 [file oncotarget-10-647-s001.pdf]

SETD2 loss sensitizes cells to PI3Kβ and AKT inhibition

SUPPLEMENTARY MATERIALS

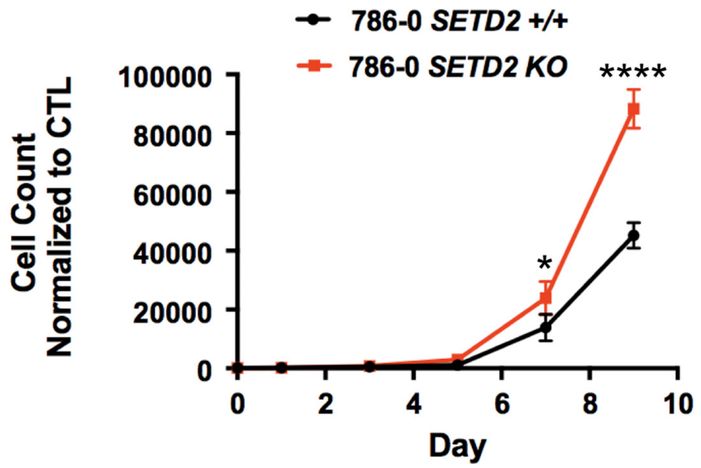

Supplementary Figure 1: Increased proliferation rate in *SETD2* deficient ccRCC-derived cells. Graph showing proliferation curves for *SETD2* proficient (+/+) and *SETD2* deficient (KO) 786-0 cells as a function of time (day). Cell count was normalized to control (CTL). $^{*}P < 0.05$; $^{****}P < 0.0001$. Standard deviations were calculated and represented for all conditions.

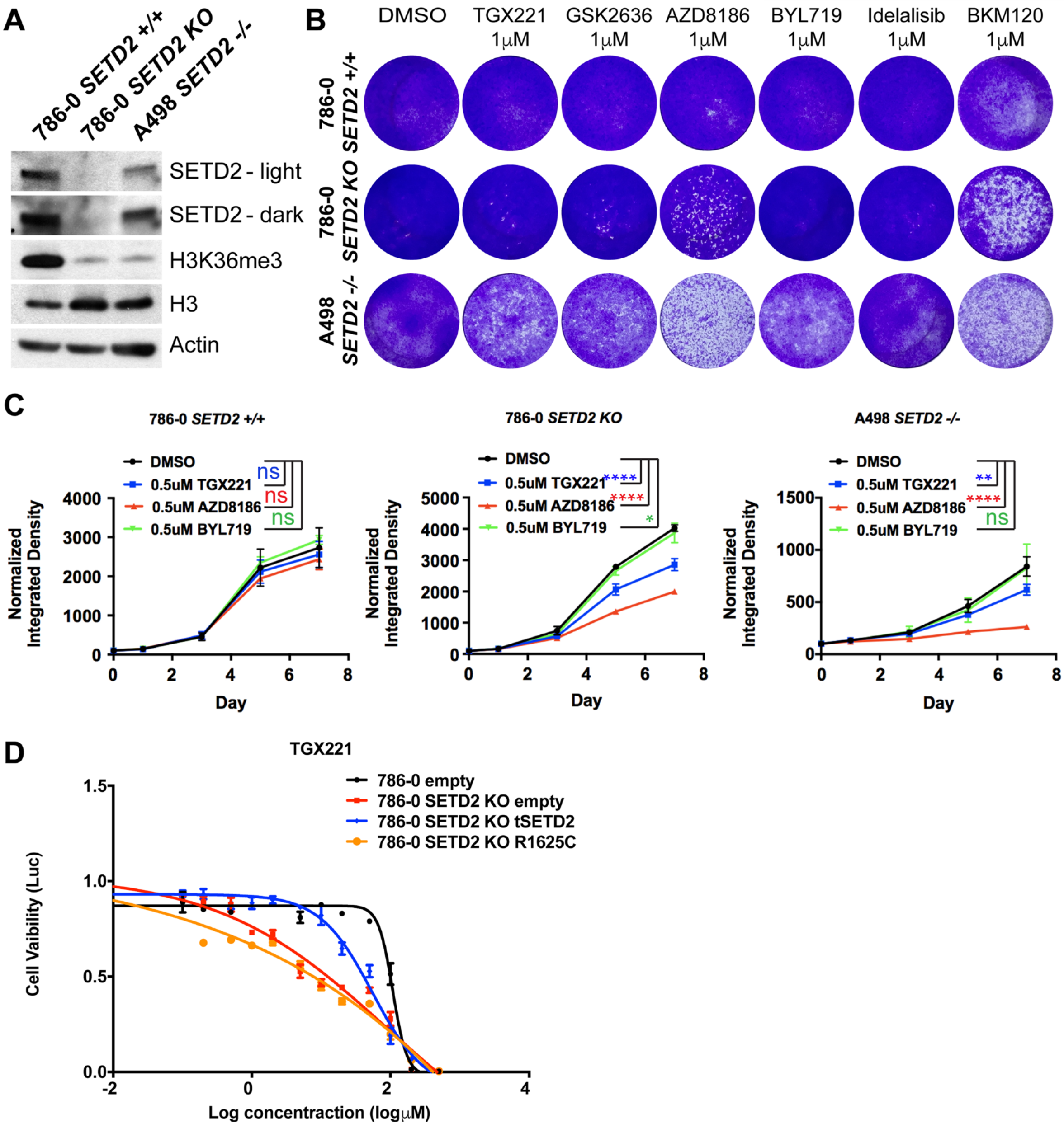

Supplementary Figure 2: Response of *SETD2* proficient and *SETD2* deficient RCC cell lines to PI3K inhibitors. (**A**) Western blot analysis of indicated proteins showing variations in histone H3 lysine 36 trimethylation (H3K36me3) levels in *SETD2* proficient (+/+) and *SETD2* deficient (KO) 786-0 and (–/–) A498 cells. SETD2 blotting is shown in two exposures (light and dark). Whole-cell protein lysates from cells grown for 24 hours were resolved by SDS-PAGE. Actin is a loading control. (**B**) Bright-field microscopy images showing living cells (attached to bottom of well) stained with 0.3% crystal violet solution of *SETD2* (+/+) 786-0 and *SETD2* (KO) 786-0 and (–/–) A498 cells were treated with vehicle (DMSO) or 1 µM inhibitor for 10 days. (**C**) Graphs plotting normalized integrated density calculated from cells stained with 0.3% crystal violet solution as a function of time (day) showing proliferation rates of *SETD2* proficient (+/+) and *SETD2* deficient (KO) 786-0 and (–/–) A498 cells treated with 0.5 µM of TGX221 (blue line), AZD8186 (red line), and BYL719 (green line) or DMSO (black line) as control for 7 days. $^{*}P < 0.05$; $^{**}P < 0.005$; $^{****}P < 0.0001$; ns, no statistical significance observed. Standard deviations were calculated and represented for all conditions. (**D**) Cell viability for 786-0 cells treated with a dose titration of TGX221. Genetic modifications to knock out and rescue SETD2 expression are indicated in the legend, showing increased sensitivity for those cells lacking SETD2 methylating activity.

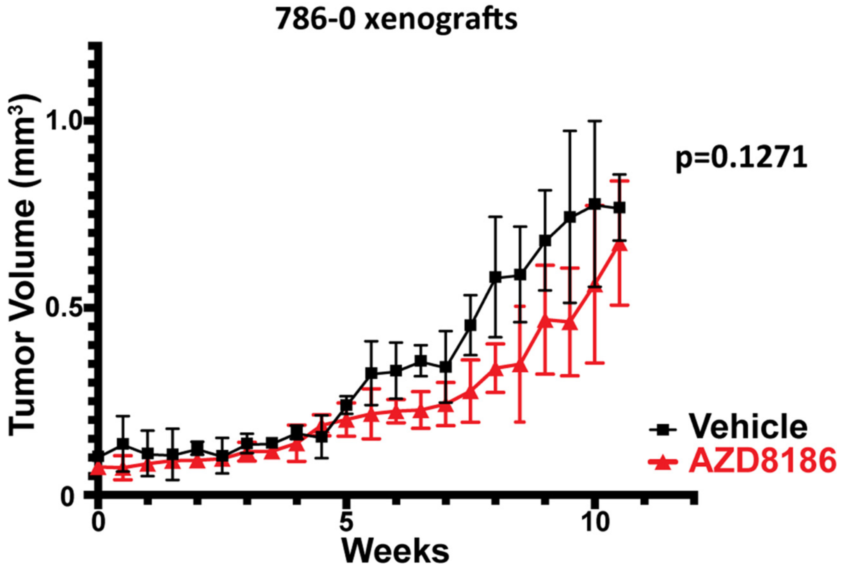

Supplementary Figure 3: PI3Kβ-specific inhibitor AZD8186 does not significantly change *SETD2* proficient tumor growth *in vivo.* Graph plotting tumor volume (mm^3) as a function of time (week) for vehicle-treated (control) and AZD8186-treated tumors. Data are represented by mean ± standard deviation.
